# Supplementary figures and images for: The splicing factor kinase, SR protein kinase 1 (SRPK1) is essential for late events in the human papillomavirus life cycle
Source: PLoS Pathog. 2025 Apr 9;21(4):e1012697. doi: 10.1371/journal.ppat.1012697 (PMC12013937; doi:10.1371/journal.ppat.1012697)

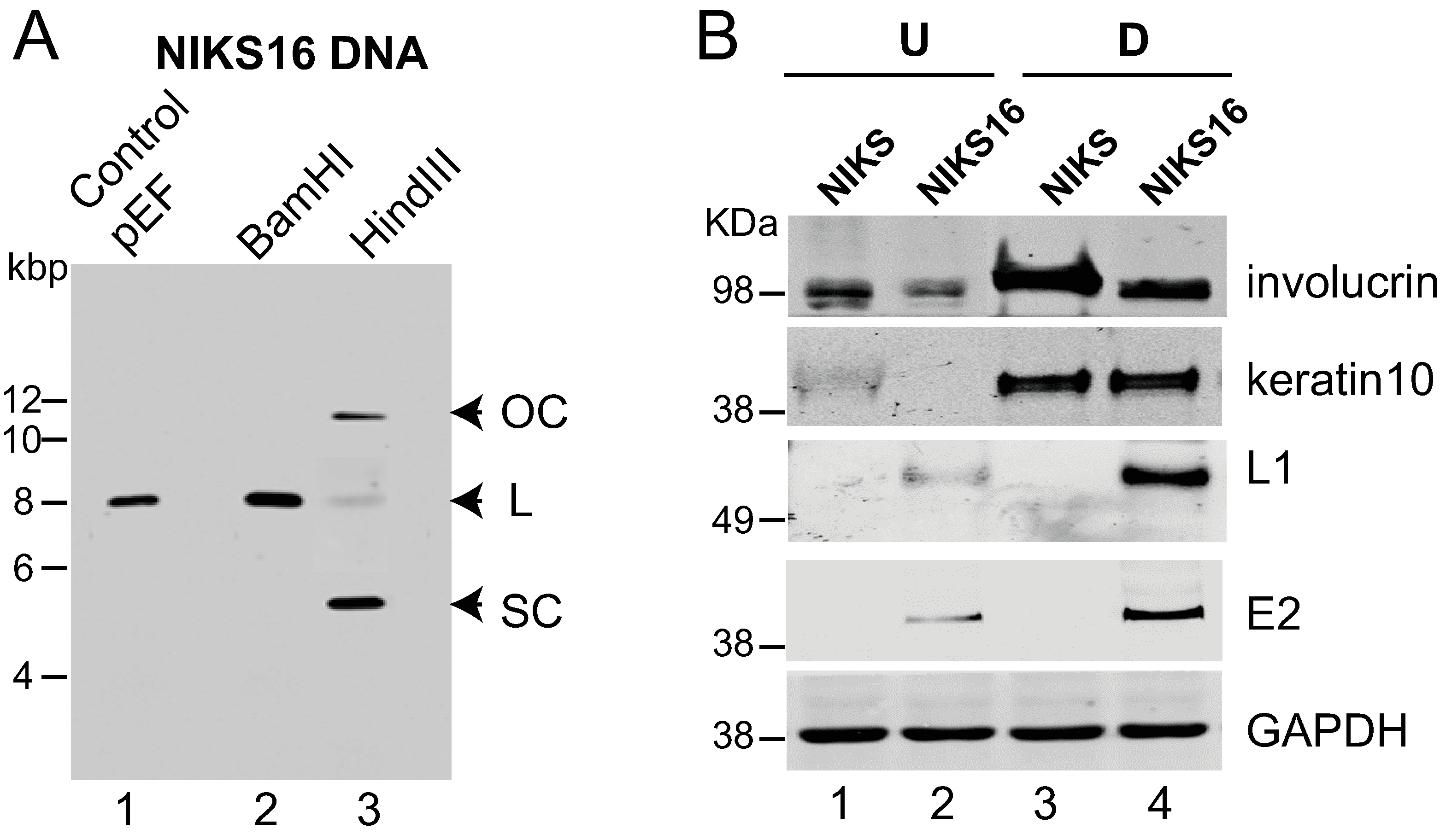

Supplement: S1 Fig — (A) Southern blot analysis of HPV16 genomes in NIKS16 cells. DNA was isolated from monolayer cultured and differentiated NIKS16 cells and digested with either Bam HI, (cuts the genome once, lane 2), or Hind III, (does not cut the genome, lane 3). The HPV16 genome was cut from plasmid pEF-HPV16 with Bam HI as a size control for the episomal genome (lane 1). (B) Western blot analysis of HPV16 E2 and L1 expression in protein lysates from HPV-negative NIKS and NIKS16 cells grown in monolayer culture to give an undifferentiated cell population (U, lanes 1 & 2) or differentiated by growing to high density in the presence of 1.2 mM Ca2+ (D, lanes 3 & 4). Involucrin and keratin 10 were used as controls for keratinocyte differentiation. GADPH is used as a loading control. (TIF) [file ppat.1012697.s001.tif]

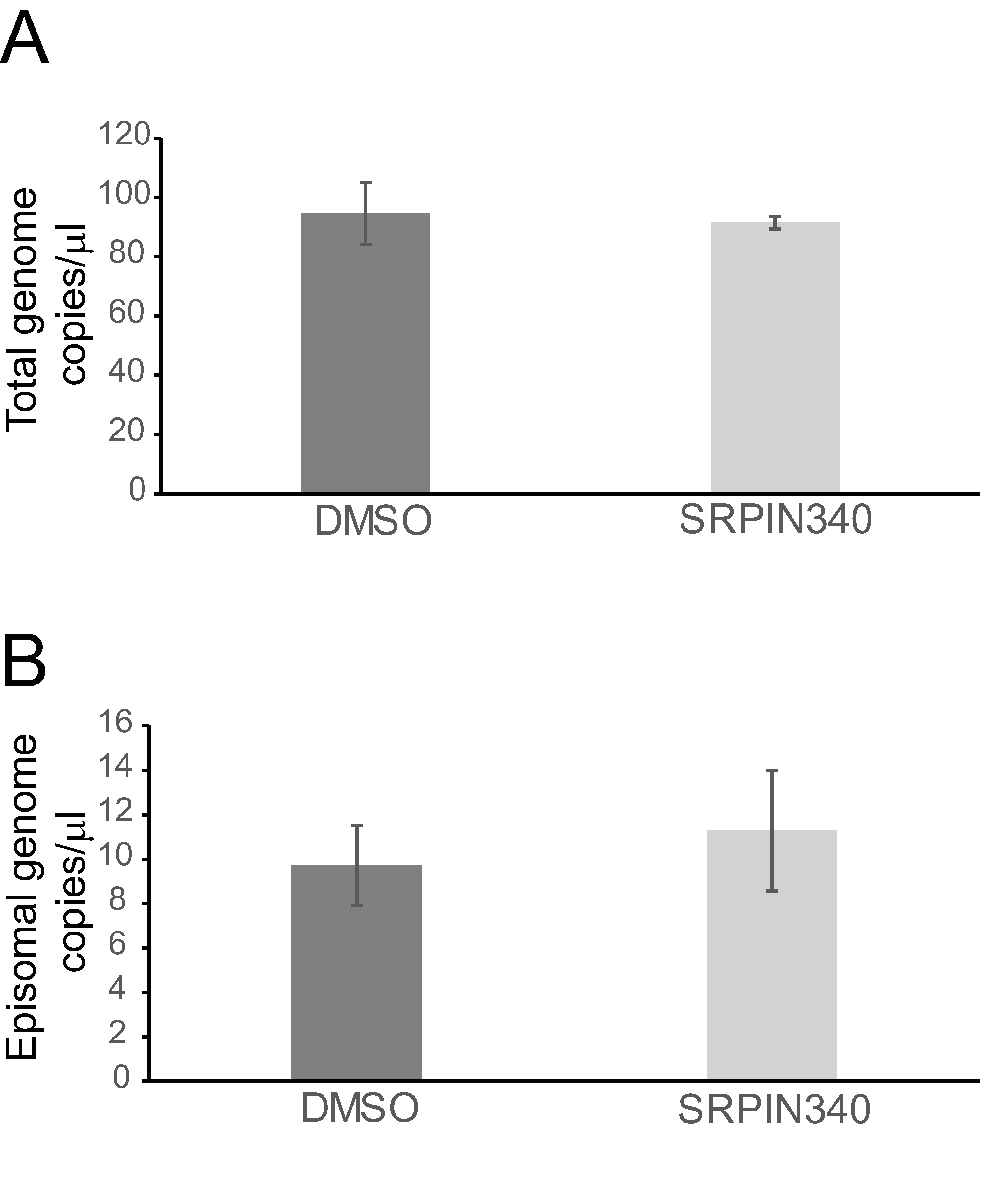

Supplement: S2 Fig — The cells used for the other experiments in the manuscript (S1 Fig) were unavailable for this experiment. The monolayer-cultured and differentiated NIKS16 cells used in this experiment had a significant number of integrated genomes. The experiment was carried out using droplet digital PCR detection of E6 gene copies. Values are shown as genome copies/µl. A. Graph showing the average number of total HPV16 genomes comparing DMSO-treated to SRPIN340-treated cells. B. Graph showing the average number of episomal HPV16 genomes comparing DMSO-treated to SRPIN340-treated cells. The data shown represent two independent experiments. (TIF) [file ppat.1012697.s002.tif]
