# Supplementary material for: The splicing factor kinase, SR protein kinase 1 (SRPK1) is essential for late events in the human papillomavirus life cycle
Source: PLoS Pathog. 2025 Apr 9;21(4):e1012697. doi: 10.1371/journal.ppat.1012697 (PMC12013937; doi:10.1371/journal.ppat.1012697)
Supplement: Attachment — Submitted filename: Response to reviewers comments.docx [file ppat.1012697.s006.docx]

**Response to reviewers’ comments**

**PPATHOGENS-D-24-02327**

**Faizo et al. “The splicing factor kinase, SR protein kinase 1 (SRPK1) is essential for late events in the human papillomavirus life cycle.”**

*We would like to thank the Editor and the Reviewers for their careful assessment of our manuscript. Their comments have helped us revise and improve the manuscript.*

**Additional Editor Comments:**

The reviewers felt that this manuscript provided interesting data to show that an inhibitor of SRPK1, SRPIN340, specifically targeted the HPV16 E2 gene expression and E2 plus E4 and L1 protein levels. To say that these changes are due to the drugs effects on SRPK1 it is necessary to show the defects in splicing of all the transcripts.

*The Editor is correct, there were no defects in splicing in any major viral transcript except for the one encoding E2. This is an important point that we failed to discuss in our first submission.* *SRPK1 does not have global effects on splicing, as we have shown here in HPV-infected keratinocytes. In experiments where the drug was applied systemically in rats, the animals remained healthy (see doi: 10.1128/AAC.00113-10.). This would not be expected if mRNA splicing was generally disrupted. SR proteins are thought to have different activity in splicing depending on the level of phosphorylation (see doi: 10.1007/s00412-013-0407-z). Therefore, inhibition of phosphorylation by SRPK1 would have different effects on different SR proteins. For HPV16, SRSF1 (an excellent substrate of SRPK1) has been shown to enhance splicing from splice donor 880 to splice acceptor 3358 to form the E1^E4 containing mRNA. However, these experiments were carried out using HPV subgenomic expression constructs in HeLa cells that overexpress SRSF1 (see doi: 10.1371/journal.pone.0072776). Our own study in NIKS12 cells containing endogenous HPV16 genomes subsequently demonstrated that SRSF1 did not have a significant role in E1^E4 mRNA expression (see doi: 10.1128/JVI.03073-15). HPV16 early gene splicing is controlled by SRSF2 (not a substrate of SRPK1) (see doi: 10.1128/JVI.03434-14) and SRSF3 (see doi: 10.1128/JVI.01719-08) (can be dynamically dephosphorylated). Therefore, splicing regulated by these SR proteins is less likely to be affected by SRPK1. SRSF9 has been shown to control late mRNA splicing in the subgenomic/HeLa cell system (see doi: 10.1099/vir.0.033183-0) but it is unknown if SRPK1 regulates this SR protein. Therefore, as you say, we cannot be certain that the changes we see are due to drug effects on SRPK1. We have added a new section to the Discussion on page 15 lines 354-370 to point this out. We have also added a new section to the Introduction to point out known differences between SR proteins and how they are phosphorylated on page 5 lines 95-100 and page 5 lines 106-111*

What accounts for the difference between early and late gene splicing?

*This is likely due to differential activity of SRPK1 on the different SR proteins and which SR proteins are required for regulation of early versus late viral mRNA splicing. We have added a comment to the Discussion on page 15 line 368 as a possible explanation of this difference.*

Throughout the manuscript each figure needs to clearly indicate which cell culture conditions are being used.

*We had already included culture conditions in most of the Figure legends. However, now for extra clarity we have added the cell culture conditions in each section of each figure legend, where missing. We have also added the exact culture conditions used for each experiment to the Results section, where it was missing.*

It is also not clear why HeLa cells are an appropriate control for NIKS16. Show controls for the E6 and E7 antibodies.

*We agree that we could have chosen a more appropriate control for NIKS16 cells, such as CaSki cells. However, HeLa cells express high levels of E6 and E7. Therefore, detection of E6 and E7 in the western blots allows unambiguous identification of E6 and E7 protein bands in NIKS16 cells where the bands are not strong. We apologise for missing out this explanation. It has now been added to the results section on page 9 line 203-205.*

**Reviewers' Comments:**

Reviewer's Responses to Questions

**Part I - Summary**
Please use this section to discuss strengths/weaknesses of study, novelty/significance, general execution and scholarship.

Reviewer #1: The authors examined the effects of an SRPK1 specific inhibitor SRPIN340 on HPV16 gene expression and life cycle in a cell line NIKS16 which harbors episomal HPV16 DNA. Experiments were conducted in submerged and differentiated (induced by high Ca++) cultures and in 3-D raft cultures. The authors also analyzed alternations in host gene expression and alternative RNA splicing based on using RNA seq data.

The authors presented some intriguing data, indicating that SRPK1 inhibited the expression of HPV16 E2 mRNA and E2 protein, as well as the production of the late E4 and L1 proteins. Interesting, the spliced E4 and L1 mRNAs were not affected. The transcription, RNA splicing and synthesis of the viral oncoproteins E6 and E7 were not affected. The authors proposed that this inhibitor could be a therapeutic target.

*We would like to thank the reviewer for their interest in our data.*

Reviewer #2: This report demonstrates that inhibition of SRPK1 disrupts viral splicing of E2 resulting in loss of E2 expression. This result supports the premise that SRPK1 inhibition is a target that could prevent HPV infections. The strengths of the paper are the potential significance of the results. The weaknesses are related to the NIKS system which is not an ideal model for HPV16 infections. However, overall there are strengths that could outweigh the weaknesses. A few points to consider from the results presented.
1. The author assignemts should be updated, a couple have no assignment. I assume that all of the work was done at UoG, therefore everyone should be assigned as UoG and current addresses can then be added.

*Thank you for pointing out this error. Assignments have been updated.*

2. Lines 175-177 state that Figs 1A-C were generated from proteins extracted from raft tissues. This is contradicted in the figure legend (lines 680-682). Please correct.

*We apologise for this mistake. The data were collected from monolayer cultured NIKS16 cells, as stated in the Figure legend. We have amended the text in the Results section on page 8 line 183.*

3. It isn't clear from this figure (1A-C) and the description what antibodies were used for what blot. pSRSF1 (and pSRSF3) while SRSF2 is listed. The legend states SRSF1 etc antibodies were used. The text is also confusing. Could the authors please clarify these figures so that they read better.

*Please accept our apologies for this confusion. All the SRSF proteins were detected with a phosphor-specific antibody. We have corrected this in the figure and clarified what was detected in the text on page 8 lines 183 and 186-188.*

4. The E6 and E7 antibody blots lack any controls, there are no non-HPV samples included. There's no need to repeat everything, but demonstrating the specificity of the bands shown with a non-HPV sample is required.

*Yes, we carried out a NIKS control for the E6 and E7 antibodies on the blots shown in Figure 2. We have prepared a new (formerly Fig 2 now Fig 3) to show entire blots in parts C and E. The text in the results section, and in the figure legend, has been changes to reflect the additional lanes in each blot.*

Also, the authors have used HPV18 HeLa extracts as a control for the antibodies. Do the authors know whether the antibodies used cross-react with 16 and 18? If they do that's fine, but stating there is more E6 and E7 in the HeLa cells should be removed as this may be due to antibody affinity.

*The antibodies do detect both HPV16 and HPV18 E6 or E7 proteins. The reviewer is correct, we cannot conclude anything about levels of E6 and E7 in NIKS16 cells relative to HeLa cells. We have now removed anything about the relative levels of E6 or E7 proteins in Hela cells compared to NIKS16 cells in the Results section.*

5. Given that SRPK1 inhibition disrupts E2 expression during differentiation the authors should determine whether viral genome integration has occurred. Loss of E2 will disrupt viral genome replication and amplification during differentiation which could result in viral integration. There's no need for Southern blots to answer this question, TV exonuclease treatment of DNA harvested from cells will easily demonstrate a trend to integration. This would also help to explain the potential disruption in other viral proteins during differentiation. The dysplastic 3D tissue following SRPK1 inhibition would support the idea that there is viral genome integration.

*If E2 is lost due to SRPK1 inhibition, then there should be a reduced probability of integration since E2 tethering of viral genomes to host chromosome is considered important for viral genome integration. We have now performed exonuclease V experiments to show that SRPIN340 treatment does not cause viral genome integration (new Supplementary Figure 2). Unfortunately, the same low passage cells used in the rest of the manuscript were not unavailable for the revision. Using higher passage cells showed significant levels of viral genome integration. However, there were still episomal genomes present and drug-treatment did not cause them to integrate. We have added a new section to the Results section to explain this and present the data on page 13-14 lines 335-344.*

Reviewer #3: The manuscript by Faizo and colleagues describes using a pharmacological approach to demonstrate the importance of the splicing regulator SRPK1 in the human papillomavirus lifecycle. More specifically they show that a small molecule inhibitor that inhibits SRPK1 activity reduces the levels of the E2 transcript and protein. E2 is a crucial virus encoded regulator of virus replication and gene expression and loss of E2 impacts on the late stages of the HPV lifecycle in a model of the virus lifecycle. RNA sequencing also shows that treatment with the small molecule inhibitor alters host cell gene expression, although it is not clear if this is only in the context of HPV infection or a broader effect. Crucially, many of the changes in gene expression correlate with targets of virus and likely counteract the de-differentiation mechanisms of the virus.

Overall this is a well written manuscript that is accessible to a wider audience. The experiments are generally well controlled and the figures are good quality.

*We would like to thank the reviewer for their positive assessment of our manuscript.*

**Part II – Major Issues: Key Experiments Required for Acceptance**
Please use this section to detail the key new experiments or modifications of existing experiments that should be absolutely required to validate study conclusions.

Generally, there should be no more than 3 such required experiments or major modifications for a "Major Revision" recommendation. If more than 3 experiments are necessary to validate the study conclusions, then you are encouraged to recommend "Reject".

Reviewer #1: There are a number of concerns as listed below:

Abstract and Introduction ( Since both sections also summarized their results and conclusions, some comments are provided under Results and Discussion)

Lines 41,42, “Drug-mediated loss of E2 is specifically associated with inhibition of late gene expression.” However, the data showed neither transcription nor splicing of early and late viral genes were affected. Only the E2 mRNA and protein, as well as E4 and L1 proteins were drastically reduced. There were no experiments to shed light on the reason for the dramatic reduction in E4 and L1 proteins. Only speculations (see Discussion section).

*The reviewer is correct. Thank you for pointing this out. We have deleted this sentence in the Abstract and the related phrase in the Introduction. We have replaced this with “**Loss of HPV E4 and L1 late proteins was also observed” on page 2 lines 40-41 and “A reduction in HPV E4 and L1 late protein levels was also observed” on page 6 lines 133-134.*

Lines 48,49, 131,132. “The treatment was not associated with changes in cell proliferation or differentiation as determined in monolayer cultures.” Please qualify the conclusion by stating "short term treatment (2 or 3 days)".

*Thank you, we have made these qualifications in the Abstract, the Introduction and the Discussion sections on* page 2 line 46, page 6 line 136, page 17 lines 427-428.

Lines 44,45, ..”the loss of…. immune function was restored”. See comments below.


Results
Line 175-179. Fig. 1 A-C : The text indicated the experiments were conducted with raft cultures. However, the Legend stated NIKS16 cells were grown as monolayer cultures. Which culture was used?

*We apologise for this error. As we reply above for reviewer 2, the data were collected from monolayer cultured NIKS16 cells, as stated in the Figure legend. We have amended the text in the Results section on page 8 line 183.*

Fig 1D-F: Clearly, monolayers cultures were used for IF, but there is no information on whether the cells were differentiated. (the same question Figs. 2 and 5, 6).

*Thank you for pointing out this omission. All experiments, except where stated otherwise, were carried out using differentiated cells. We apologise that we did not explicitly state this throughout the Results text and Figure legends. We have now inserted the word “differentiated” throughout the Results text and in the Figure legends, where appropriate.*

Line 175-179, the statement “SRSF1 and SRSF3 were found in the cytoplasm” is overstated; it appears that only portions of each protein were in the cytoplasm. Nuclear localization cannot be ruled out in the absence of subcellular fractionation.

*The reviewer is completely correct. We apologise for our inaccuracy. We have changed the text on page ….to read “SRSF1 and SRSF3 were found in both the nucleus and the cytoplasm in SRPIN340-treated NIKS16 cells” on page 8 line 191.*

Line 142-Fig. 3 Legend, HPV16 "late" promoter….. should be "early" promoter.

*We have not shown the early promoter in this diagram (now Figure 4A) since we are focussing on late gene expression. We have shown only the late promoter at nt 670.*

Line 266-270. “ One important known change to the epithelium caused by HPV 16 infection is an abrogation of keratinocyte differentiation and loss of both structural and immune aspects of the epithelial barrier.” This statement is not entirely correct and should be rephrased. The virus life cycle is dependent on epithelial differentiation (which the author also stated in the Introduction). Indeed, the involucrin expression is not affected as shown in the Figs. 3 and 4. As far as I am aware of, keratin 10 was not abrogated either.

*We have deleted the phrase in the text and replaced it with “Previously we showed that HPV16 infection leads to loss of both structural and immune aspects of the epithelial barrier”. The use of the word abrogate was a mistake.*

Line 274,275: “SRPIN340 affected the expression of 5 genes encoding key immune regulators, including IRF1 (Fig .5E), which are known to be modulated by HPV16 infection.” Fig. 5E, shows that he HPV16 infected cells down-regulated 3 immune response genes, while upregulating 3 others, including IRF1.

*We apologise for our inaccuracy. We have corrected the text from 5 to 6 genes on page 12 line 285-286.*

The figures also shows that SRPIN340 had the opposite effects on these 6 genes, including down-regulating IRF1. However, HPV oncoproteins are known to suppress immune response genes, including IRF1. (a similar concern for Statements in Discussion section as well).

*In a previous publication we showed that HPV16 infection up-regulated IRF1 in differentiated NIKS16 keratinocytes (doi: 10.1128/JVI.01001-17.). This agrees with another transcriptomic study where IRF1 was shown to increase in expression in HPV16-infected compared to uninfected cervical keratinocytes (see* doi: 10.1128/JVI.00915-19). *Data by others have indeed shown that HPV oncoproteins downregulate IRF1. However, these data were obtained using oncogene overexpression experiments in cells, or in mice overexpressing E7 in the epithelium, or in tumour cells, or in HPV-associated cancers.*

Line 296-299, “Splicing of transcripts encoding DNA repair factors/DNA damage response was also significantly altered (Fig 5F). The DNA damage response is essential for HPV replication and alternative splicing-induced changes in DNA repair factors could create a cellular environment inhibitory to HPV replication.” This statement is an overreach without validation. It should be deleted. First of all, the loss of viral E2 protein would have incapacitated viral DNA replication. Secondly, the authors never examined viral DNA replication in inhibitor treated or control untreated raft cultures. Lastly, there are many DNA repair factors. The authors did not provide any information on the identity of these host factors with altered splicing. Therefore, it is not possible to know whether these factors are known to be required for viral DNA replication.

*Thank you for pointing this out. We have deleted the second sentence but have retained the first one since this is what our data show. (see page 12 last line).*

Fig .6. The author statement in the Discussion that they studied only differentiated NIKS16 in submerged cells. But this cannot be the case, as they can only examined cell proliferation and viability by using proliferating cells. It is recommended that authors explicitly specify the culture conditions for each experiment in the text and in the Fig. Legends.

*Thank you for pointing out this error. We have now corrected and specified all the culture conditions in the Results text and in the figure legends.*

In Fig. 6. The inhibitor appeared to reduce the cell number and viability at 72 hr. but the authors considered it insignificant. Only longer treatment can reveal whether the cell number and viability continued to trend down.

*The reviewer is correct. We have inserted a disclaimer statement in the text “However, we cannot exclude a significant change to cell proliferation or viability at later time points.” on page 13 lines 332-333.*

Discussion
This section was long and much was just speculation. It should be revised and shortened. Some examples and additional minor points are presented below.

*We have shortened the Discussion but have inserted a new second paragraph on page 15 to address the main comment of the Editor. We have followed the reviewer’s recommendations as stated below.*

Line 325,”SRPIN340 specifically inhibits alternative splicing of the E2 mRNA”. It is more accurate to state “…inhibits the splicing which produces the E2 mRNA.”

*We have made this change on page 15 line 352.*

The authors speculated perhaps SRSF5 (and hnRNP G) might play roles in generating the E2 mRNA. Did the authors detect any changes in SRSF5 expression at the level of RNA or protein?

*Yes, we did this using siRNAs against all the classical SR proteins but only one experiment was performed. This does not allow us to conclude anything about significance. We have deleted this section since it is speculation.*

Line 342-343, “SRPK1 may be involved in promoting viral DNA replication, genome transcription, and mRNA splicing.” This statement is not completely accurate. The authors data show that the viral genome transcription was not affected, only the RNA splicing to produce the E2 mRNA was. SRPK1 is involved in promoting viral DNA replication, but only indirectly through its effect on RNA splicing to generate the E2 mRNA, hence protein.

*The reviewer is correct. We have deleted this statement.*

Line 351-352 “ .. E2 can bind both E4 and L1 proteins [46, 47], could cause a reduction in levels of both proteins at a post-transcriptional level or perhaps by destabilising the proteins. This is purely illogical speculation. Since there is little or no E2 protein, how would it destabilize the late proteins? Did the author meant to say, the lack of E2 would destabilize the late viral proteins? This is very unlikely either. Chances are the early phase will not overlap the late phase for a simple reason. it is not possible to encapsidate viral DNA while it is undergoing replication. As to E4, it is the most abundant viral protein and is cytoplasmic, not nuclear as the E2 of low abundant. Their “transient interaction if any, or lack of it could not have affected the level of E4 protein.

*We have rewritten this section to delete the above as it is speculation. However, others have shown that capsid proteins are present in cells expressing E2. For example, E2 may be involved in capsid assembly. It has been proposed that L2 interacts with E2 to inhibit viral transcription.  E2 also interacts with L1 at viral replication foci to enhances transcription and replication of viral genomes (see doi: 10.1002/jmv.29461 for a summary). E2 can be found in the nucleus and the cytoplasm (see doi: 10.1099/vir.0.000162).*

Line 355- 358, “Inhibition of late events in the viral life cycle could imbalance the differentiation-dependent life cycle and potentially result in persistent infection and cancer progression. However, we found no evidence for any tumour-promoting activity of SRPIN340 in HPV16-positive keratinocytes, since cell proliferation and viability were unchanged in the presence of the drug, and repression of differentiation due to HPV infection was reversed. Importantly, we found that E6E7 expression and activity were not enhanced by drug treatment. “ . A 2 day treatment in raft cultures and 3-day treatment on submerged cultures cannot be used to predict its potential to promote cancer progression. This statement should be deleted.

*We agree with the reviewer and have removed this statement.*

Line 372-374, “This drug induced effect would be predicted to mechanically strengthen infected, differentiated keratinocytes and inhibit HPV virion egress from the surface of the epithelium.” This sentence is partially true. However, there will not be virus production for lack of L1 and for the inability to replication DNA in the absence E2.

*The reviewer is correct. We have removed this section.*

Line 393-396,” Therefore, although we have studied SRPIN340 activity in the HPV16 life cycle, it may have potential in therapeutic treatment of HPV-associated cancers. Cancer-specific splicing can lead to the expression of oncogenic protein isoforms, as has been shown for HPV-positive HNSCC [51, 52], which SRPIN340 could ameliorate.” This manuscript did not address the issue whether the inhibitor can alter cancer-specific alternative splicing in host genes. The statement should be deleted.

*Others have shown clearly that SRPIN340 can change VEGF-A splicing to non-oncogenic forms. VEGF-A inhibition is a current second-line treatment for cervical cancer. We feel it is important to highlight this fact. We have rewritten the paragraph and have deleted the second sentence above. See page 17 lines 411-416.*

Reviewer #2: 1. Demonstrate that the oncoprotein antibodies have specificity by running a non-HPV control side by side with HPV16 samples.

*We have replaced the panels in Figure (now Figure 4) to show the NIKS non-HPV controls.*

2. Determine whether SRPK1 inhibition promotes viral genome integration due to the disruption of E2 expression. This is important as integration is often associated with progression to cancer.

We have now carried out this experiment and show the data in Supplementary Figure 2as explained above.

Reviewer #3: (No Response)

**Part III – Minor Issues: Editorial and Data Presentation Modifications**
Please use this section for editorial suggestions as well as relatively minor modifications of existing data that would enhance clarity.

Reviewer #1: The manuscript needs a thorough read to improve writing and clarity. For instance, there are many ….that…….that…; …that… which; …. whose….which….. Long and complex sentences should be split into sperate sentences.

*We have rewritten the manuscript to divide the long and complex sentences into separate parts. The majority of “that” “which” and “and” in clauses have been removed. However, addition of the phrases to specify the various culture conditions as requested by the editor has required some complex sentences. We hope we have improved the clarity throughout.*

Reviewer #2: See above.

Reviewer #3: In this reviewer's view the supplementary experiments should be moved (if possible) into the main manuscript as they are necessary for understanding the early section of the results chapter. Having them in the main body will help the reader.

*Thank you for this suggestion. We have now placed Supplementary Figures 1 and 3 in the main body of the manuscript. Supplementary Figure 2 is simply an assessment of the NIKS16 model system. We have retained this as a Supplementary Figure.*

Fig1D - in the DMSO merge it looks like there might be an issue with the image. It appears as though some of the cell are obscured by something. Has this image been manipulated?

*We have examined this panel at high magnification. We cannot see anything obscuring any of the cells in the image. However, to avoid any issues, we have replaced the image with an alternative one.*

Fig 2 – it is not clear to me why the authors chose to use HPV18 positive HeLa cells as the control for E6/E7 expression in these experiments rather than a HPV16 positive cancer cell line.

*We agree that it was a bad decision to use HeLa cells. It would have been better to have used SiHa or CaSki cells. However, the HeLa cells still allow verification of the specificity of the E6 and E7 antibodies.*

Also, in the graphs it might be better to normalise to the NIKS16 mock rather than the cancer cells, which have much higher levels of oncogene expression. This might make it easier to interpret the data.

*We would like to thank the reviewer for this good suggestion. We have changed the graphs In Fig 2 (now Fig 3) accordingly.*


Fig 3 - These changes are not essential but the data in fig 3 could be strengthened through alternative approaches such as shRNA knockdown of SRPK1 or introduction of DN kinase mutations.

*We agree with the reviewer that this would be helpful to strengthen our data. Unfortunately, the student who carried out the work left several years ago. It would take many months to carry out shRNA depletion experiments. In addition, drug inhibition is a more specific way to inhibit the kinase since there is no risk of off-target effects. Creating and testing a dominant negative SRPK1 mutant would take even longer and there is no one in the lab to carry out this work.*

Fig 4 – The western blots and staining of the rafts show a very clear reduction in late viral protein expression. What is the mechanism for this given that no major differences in late transcript levels were seen in the previous figure?

*This is an interesting point that we have address on page 16 lines 382-387 of the Discussion.*

Fig 5 – how do the gene expression changes relate to the previous studies showing E2 regulates gene expression changes?

*We had carried out a comparison of the data from our study with that of the Morgan group who examined E2-induced transcriptome changes in N/Tert-1 immortalised foreskin keratinocytes. Only 27 genes were up-regulated in SRPIN340-treated cells and down-regulated in E2 overexpressing cells. The N/Tert-1-E2 cells overexpress hTERT and E2. In contrast, NIKS16 cells express hTERT and E2 at endogenous levels. Since E2 is a transcription factor that controls cellular genes, and hTERT overexpression can promote oncogenesis, this could lead to the lack of similarity.*

Fig 5 - it would be important to confirm some of the gene expression changes seen in the RNA sequencing at protein level in your model systems. In particular it would strengthen the manuscript to show that levels of differentiation markers have been altered. Would you have anticipated being able to see changes in morphology of the treated rafts given the RNA-seq data?

*We carried out the differentiation marker experiments already. We have now included these in a new figure, Figure 8. The figure shows 3D raft cultures immunohistochemically stained for differentiation markers involucrin and keratin 10. We have added a new section to the Results section on page 13 lines 308-316. It would be very difficult to detect changes revealed by RNASeq simply by looking at morphology.*

What happens after longer term treatment of the drug? Do you see a greater impact on NIKS16 proliferation?

*We did not carry out longer term treatment with the drug in the monolayer cultured cells. Since we have differentiated the cells, the colonies formed overgrow each other and cells begin to die after 10 days in culture.*
